# Editorial: Artificial Intelligence for eHealth

**DOI:** 10.3389/fpubh.2022.852840

**Published:** 2022-03-04

**Authors:** Deepak Gupta, Joel J. P. C. Rodrigues, Sheng-Lung Peng, Nhu Nguyen

**Affiliations:** ^1^Department of Computer Science and Engineering, Maharaja Agrasen Institute of Technology, New Delhi, India; ^2^Senac Faculty of Ceará, Fortaleza, Brazil; ^3^Department of Creative Technologies and Product Design, National Taipei University of Business, Taipei, Taiwan; ^4^Duy Tan University, Da Nang, Vietnam

**Keywords:** artificial intelligence (AI), eHealth, computational intelligence, COVID-19, neural network

Artificial intelligence has grown extensively in recent times and is changing the healthcare industry from many perspectives: Clinical Diagnosis, suggests treatment and follow up. Clinical Decision Support (CDS) is a major topic of AI in medicine to assist clinicians at point of care. Existing techniques used for processing health data can be broadly classified into two categories: (a) non-Artificial Intelligence (AI) systems and (b) Artificial Intelligence systems. Even though non-AI techniques are less complex in nature, most of the systems suffer from the drawbacks of inaccuracy and lack of convergence. Hence, these systems are generally replaced by AI based systems which are much superior to the conventional systems. AI techniques are mostly hybrid in nature and include Artificial Neural Networks (ANN), fuzzy theory, and evolutionary algorithms. AI increases the ability for healthcare professionals to better understand the day-to-day patterns and needs of the people they care for, and with that understanding they are able to provide better feedback, guidance and support for staying healthy.

AI-based CDS uses inference and logics, while non-AI-based CDS relies on machine learning to perform the same functions. There are many clinical duties that CDS may assist with, but it is essential that CDS is correctly integrated into the clinical workflow and health records. CDS can be used to assist clinicians in the interpretation of medical pictures through the use of Computer Aided Diagnosis (CAD). CAD incorporates AI as well as computer vision, signal processing, and other components relevant to medicine. Breast cancer, lung cancer, colon cancer, coronary artery disease, and Alzheimer's disease are just a few of the conditions that can benefit from CADs.

There are certain societal concerns about the expanding use of AI in healthcare, including the possibility of bias, lack of transparency for some AI algorithms, privacy problems for data used for AI model training, and security and implementation responsibilities in clinical settings.

All areas of artificial intelligence (AI) in the fields of health informatics, biomedical informatics, and medical image analysis are covered in this special issue. Based on the reviews, eight papers were chosen from a total of fifteen submissions to this special collection. At least two reviewers and at least two rounds of review were required for each paper. Listed below are some of the papers that made important contributions to this discussion.

Using data from the Alzheimer's Disease Neuroimaging Initiative (ADNI) and independent latent class analysis (LCA and LTA) and latent transition analysis (LTA) over a three-year period, the authors (Alashwal et al.), describe their findings in the first paper of this special issue. Researchers found that LCA was a better predictor of AD progression than typical clinical cut-off measures on neuropsychological exams when it came to defining and recognizing the disease.

Researchers (Mishra et al.) recommend data preparation to eliminate the problem of data skewing by employing sample approaches. The K-Nearest Neighbor algorithm is used to classify the data using three different sampling techniques: Resampling, Spread Subsampling, and SMOTE. Evaluation of classification's performance is done using a variety of performance indicators to determine classification's efficiency.

The authors (Iwendi et al.) in this research offer a fine-tuned AdaBoost algorithm-boosted Random Forest model. Data from the COVID-19 patients is used to create a model that can estimate the severity of a patient's condition and whether or not they will recover or die. On the dataset used, the model has an accuracy of 94% and an F1 Score of 0.86. The study of the data shows a link between the gender of the patients and their mortality, as well as the fact that the vast majority of patients are between the ages of 20 and 70.

They are trying to find out if heat massaging of the spinal column can reduce muscle discomfort and increase antioxidant function in this study (Kim et al.). There were 60 people in the study who had lower back discomfort. Both an experimental and a control group were given spinal column heat massage and normal rehabilitative treatment, respectively, as part of their rehabilitation. According to the results of the study, spinal column thermal massage decreases pain more efficiently and improves impairment levels. Because of this, thermal massage may be beneficial in the treatment and prevention of oxidative disorders.

In the next paper (Song et al.) of this special collection shows that the pectolinarin triggers apoptotic cell death in PC12 cells by DNA fragmentation and the production of apoptotic bodies via the activation of ER stress sensors (eIF2 phosphorylation and ATF6 fragmentation) in PC12 cells. The treatment of PC12 cells with 50 μM pectolinarin for 24 h increased the mRNA expression of ATF6, PERK, and IRE1 by up to 1.6, 1.7, and 1.4 times, respectively, compared to the control. Pectolinarin administration enhanced ATF6 fragmentation by roughly twofold compared to the control, and phosphorylation of eIF2 by 2.5 fold. As a result of these findings, future natural medicines and health supplements targeting disorders caused by apoptosis could benefit from a better understanding of the molecular pathways involved.

For the purpose of estimating the number of people who will die from COVID-19-related causes in India over the next decade, the authors (Dhamodharavadhani et al.) conducted an investigation into the suitability of Statistical Neural Network (SNN) models and their hybrid version. These SNN models, including the Probabilistic Neural Network and a Radial Basis Function Neural Network, are used to construct the COVID-19 Mortality Rate Prediction model (MRP) in India. MRP models based on PNN and RBFNN were found to perform better than other models in COVID-19 datasets D2 and D1.

Based on an autoregressive integrated moving average, a model was developed to predict an epidemic of COVID-19 in the world in the next several days (ARIMA) (Dansana et al.). In addition to the 120,000 confirmed fatalities predicted by the ARIMA model until April 1, 2020, we also evaluated the total number of confirmed cases, the total number of fatalities predicted, the autocorrelation function, and the white noise time series for the COVID-19 outbreak's confirmed and fatalities cases.

Patients infected with the COVID-19 virus can have their medical issues diagnosed using a data mining model built on a hybrid deep learning framework (Khadidos et al.). Convolution neural networks (CNNs) and recurrent neural networks (RNNs) combine to form the DeepSense technique, a hybrid deep learning model. In comparison to other deep learning and machine learning classifiers, DeepSense's accuracy was shown to be significantly higher. A patient's prognosis for COVID-19 infections can be improved by knowing the accuracy of the diagnostic approach used.

To summarize, eight of the fifteen papers submitted to this special issue were accepted for publication in this special edition. We, as guest editors, believe that this special issue's research contributions and conclusions will assist readers by expanding their knowledge and inspiring them to work on a variety of elements of Artificial Intelligence for eHealth themselves.

## Author Contributions

All authors listed have made a substantial, direct, and intellectual contribution to the work and approved it for publication.

## Conflict of Interest

The authors declare that the research was conducted in the absence of any commercial or financial relationships that could be construed as a potential conflict of interest.

## Publisher's Note

All claims expressed in this article are solely those of the authors and do not necessarily represent those of their affiliated organizations, or those of the publisher, the editors and the reviewers. Any product that may be evaluated in this article, or claim that may be made by its manufacturer, is not guaranteed or endorsed by the publisher.

